# A striking case of pigmented demodicosis

**DOI:** 10.1093/skinhd/vzaf054

**Published:** 2025-07-22

**Authors:** Shanxi Jen, Lydia Tang-Lin, Sze Hwa Tan, Wei Liang Koh

**Affiliations:** Department of Dermatology, Changi General Hospital, Singapore, Singapore; Department of Dermatology, Changi General Hospital, Singapore, Singapore; Department of Anatomical Pathology, Changi General Hospital, Singapore, Singapore; Department of Dermatology, Changi General Hospital, Singapore, Singapore

## Abstract

This case report presents a rare occurrence of pigmented demodicosis in a 59-year-old woman with Fitzpatrick skin type IV, who presented with pruritic and progressively pigmented plaques over her cheeks and eyelids. Dermoscopic examination and skin biopsy confirmed the presence of *Demodex* mites in the hair follicles. Treatment with topical ivermectin led to marked improvement, highlighting the importance of considering this condition in cases of facial hyperpigmentation and the efficacy of antidemodectic therapies.

## Teaser

This case report presents a rare occurrence of pigmented demodicosis in a 59-year-old woman with Fitzpatrick skin type IV, who presented with pruritic and progressively pigmented plaques over her cheeks and eyelids. Dermoscopic examination and skin biopsy confirmed the presence of *Demodex* organisms in the hair follicles. Treatment with topical ivermectin led to marked improvement, highlighting the importance of considering this condition in cases of facial hyperpigmentation and the efficacy of anti-demodectic therapies.

**What is already known about this topic?**

- *Demodex* mites have been predominantly implicated in inflammatory cutaneous conditions such as folliculitis, rosacea, seborrhoeic dermatitis and blepharitis.
- There are limited reports linking demodicosis to facial hyperpigmentation in the published literature.

**What does this study add?**

- The rarity of pigmented demodicosis and the limited literature available on this topic underscore the significance of this case report.
- Our aim in sharing this case with the medical community is to increase awareness and improve the recognition of this specific variant of demodicosis, along with its potential predisposing risk factors, such as diabetes mellitus and chronic kidney disease.

## Case report

*Demodex* mites have been predominantly implicated in inflammatory cutaneous conditions such as folliculitis, rosacea, seborrhoeic dermatitis and blepharitis. There have been infrequent links of demodicosis to facial hyperpigmentation in published literature. In this case report, we demonstrate a rare case of pigmented demodicosis.

**Figure 1:**
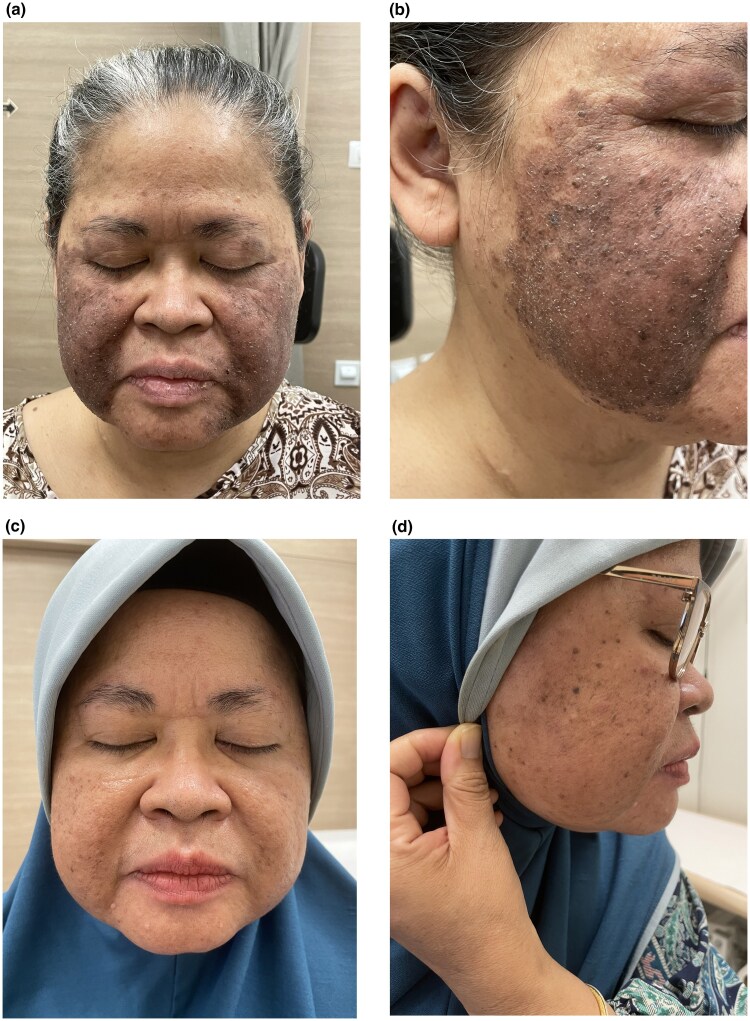
Clinical pictures of the face and cheek of a 59-year-old Malay woman with pigmented demodicosis before and after treatment. (a) Initial presentation, front view; (b) initial presentation, side view; (c) 7 months after treatment with topical ivermectin 1% cream once daily, front view; (d) 7 months after treatment with topical ivermectin 1% cream once daily, side view.

**Figure 2:**
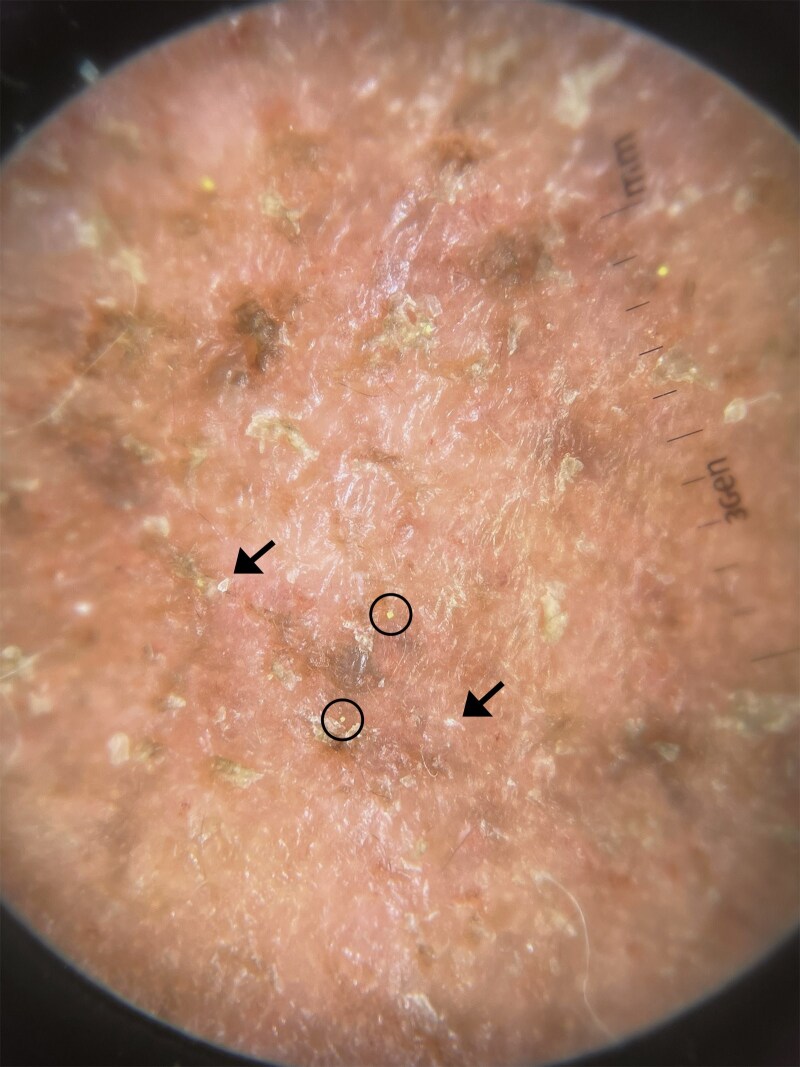
Dermoscopic image of the left cheek. Dermoscopic image (magnification ×10) showing conical-shaped filaments protruding from follicular openings suggestive of *Demodex* ‘tails’ (circles) and follicular scaling (arrows), on a background of non-specific brownish-yellow scale-crust.

A 59-year-old Malay woman, with Fitzpatrick skin type IV, presented with a 6-month history of an intermittently pruritic initially erythematous rash over her cheeks which became increasingly pigmented over time. Her past medical history included type 2 diabetes mellitus, hyperlipidaemia, hypertension and end-stage renal failure on haemodialysis. Physical examination revealed prominent well-demarcated slightly greasy-looking brown–red plaques with fine follicular scaling over bilateral cheeks and lateral aspect of bilateral upper eyelids, sparing the nasolabial folds and nose (Figure 1a, b). Dermoscopic examination revealed white conical-shaped filaments protruding from follicular openings suggestive of *Demodex* ‘tails’, a finding specific for demodicosis, and nonspecific superficial scales (Figure 2). A clinical diagnosis of pigmented demodicosis was made. Differential diagnoses included seborrhoeic dermatitis, contact dermatitis and pemphigus foliaceus/erythematosus. Fungal scrape for microscopy and culture were negative.

**Figure 3:**
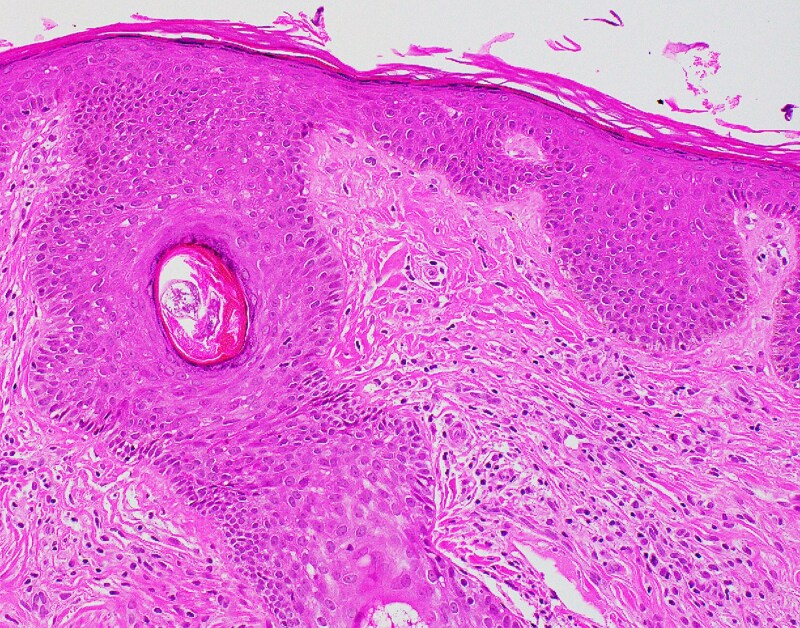
Histopathology sample of skin punch biopsy from left cheek. Haematoxylin and eosin (magnification ×200): high-power view demonstrating *Demodex* organisms in one of the hair follicles. The dermis shows a mild lymphohistiocytic infiltrate admixed with a few eosinophils around the superficial dermal vessels.

A skin-punch biopsy was performed over the left cheek and showed mild acanthosis, spongiosis and focal hyperkeratosis. A mild lymphohistiocytic infiltrate admixed with a few eosinophils was present around the superficial dermal vessels. Notably, *Demodex* organisms were identified in the hair follicle structures (Figure 3). There were no interface changes or obvious acantholysis. Antidemodectic treatment with topical ivermectin 1% cream once daily was initiated and led to improvement of hyperpigmentation within 1 month of follow-up. Maintenance therapy with topical ivermectin cream was advised. Seven months after initiation of treatment, the patient experienced further reduction in hyperpigmentation and resolution of the greasy appearance and scaling (Figure 1c, d).

## Discussion

Two *Demodex* species have been found to inhabit the pilosebaceous unit in humans: *Demodex folliculorum* and *Demodex brevis*. Chiefly subsisting on sebum components and epidermal cells, *Demodex* mites are found in greater concentration in areas rich in sebaceous glands such as the face, cheeks, forehead, and Meibomian glands of the eyelids.^1^ Although the presence of *Demodex* mites are often asymptomatic, they can cause both dermatological and ophthalmological conditions when the equilibrium balancing the skin’s microenvironment, the density of *Demodex* mites and one’s immune system is disrupted.^1,2^ Factors such as immunosuppression, systemic diseases such as diabetes mellitus and chronic kidney disease (as seen in our patient), and sebaceous gland hyperplasia have been implicated in promoting the proliferation of *Demodex* mites.^2–4^ Common dermatological symptoms include pruritus, erythema, and rough or scaly skin.^1,3^ Demodicosis manifests when concentration of *Demodex* mites increases in the sebaceous unit (density of >5 cm^2^)^2^ or upon penetration of mite into the dermis.^1,3^ In our patient, we found a few mites within the follicles seen within one section of a 4-mm skin-punch biopsy.

Predominantly recognized in inflammatory cutaneous conditions such as folliculitis, rosacea and seborrhoeic dermatitis, demodicosis has only been rarely linked to hyperpigmentation.^5^ Postinflammatory hyperpigmentation may occur following mechanical damage and local inflammatory processes incited by mites in the hair follicles, which eventually cause damage to epidermal cells. Diagnosis involves relevant clinical symptoms, high density of *Demodex* mites, and clinical resolution of signs and symptoms after acaricidal treatment.^2^ Standardized skin surface biopsy and/or direct microscopic examination can be offered to exclude other differentials and to determine demodex mite density. Treatment for demodicosis has not been standardized. A recent meta-analysis of 21 studies by Li *et al.*^6^ found ivermectin (topical and systemic), ivermectin–metronidazole (topical) and tea tree oil (topical) effective as antidemodectic therapies, with reported side effects being predominantly mild.

This case report highlights pigmented demodicosis as a differential diagnosis for facial hyperpigmentation. It is important to be cognizant of this under-recognized condition as initiation of appropriate antidemodectic treatment can lead to significant improvement.

## Data Availability

The data underlying this article are available in the article.
